# Supplementary material for: Chromothripsis during telomere crisis is independent of NHEJ, and consistent with a replicative origin
Source: Genome Res. 2019 May;29(5):737–49. doi: 10.1101/gr.240705.118 (PMC6499312; doi:10.1101/gr.240705.118)
Supplement: Supplemental Material [file supp_gr.240705.118_Supplemental_file_1.zip › contigs/annotated_contigs/DB80/contig.2.DB80_length_444_mean_cov_11.8243243243.docx]

**DB80_length_444_mean_cov_11.8243243243**

```
TTAAAATCAACTCAATTTACCCTCTTGTTTTAAGAAATTCTTAACAATTGCCTTCCACATTGTCAAAAACAGCTAGGATTTAACTGGAA
 >chr7:110766836-110767136 - E=4e-169 p=3e-02
ACTGTCCATGCTATCACAAAATAAATGTCTAATATTAACAAATAAATTGATATGACTACAGTGCTGGAAATGTTTATAGGCCAGTGAGA

TGTTGATATTATGCAGAAAGCTATTGTAAGAAAAGTTAAAAGTTGCTTATGAGCAGACACAGTAGCAATTAACATGAAAGCTTTGTAAC

TTTGAGAGATAAACAATTTGCCTATAAAAA|CTG|CATAAATGTCTTCTTTTGAGAATTGTCTGTTCATATCCATGTCTAAAACACCAA
                               >chr7:110761368-110761515 - E=6e-77
AAGCAATGGCAACAAAAGCCAAAATTGACAAATGGGATCTAATTAAACTAAAGAGCTTCTGCACAGCAAAAGAAACTACCATCAGAGCG

A
```
